# Learning robot differential movements using a new educational robotics simulation tool

**DOI:** 10.1007/s10639-022-11433-6

**Published:** 2023-02-20

**Authors:** Fernando Gonzalez

**Affiliations:** Department of Computing and Software Engineering, Florida Gulf Coast University, 10501 FGCU Blvd. South, Fort Myers, FL 33965 USA

**Keywords:** Robotics, Education, Differential movements, Robot joint programming, Virtual robot, Robot programming, Introduction to robotics course, Robot simulation

## Abstract

The study of robotics has become a popular course among many educational programs, especially as a technical elective. A significant part of this course involves having the students learn how to program the movement of a robotic arm by controlling the velocity of its individual joint motors, a topic referred to as joint programming. They must learn how to develop algorithms to move the end effector of the arm by controlling the instantaneous velocity or some similar aspect, of each joint motor. To support this learning activity, physical or virtual robotic arms are typically employed. Visual observation of the movement of the arm provides feedback to the correctness of the student’s joint programming algorithms. A problem arises with supporting the student in learning how to move the robotic arm with precise velocity along some path, a subtopic of joint programming referred to as differential movements. To develop this knowledge, the student must produce and test differential movement algorithms and have the capability to verify its correctness. Regardless of the type of arm used, physical or virtual, the human eye cannot notice the difference between a correct or incorrect movement of the end effector as this will involve noticing small differences in velocities. This study found that by simulating the process of spray painting on a virtual canvas, the correctness of a differential movement algorithm may be accessed by observing the resulting paint on the canvas as opposed to observing the movement of the arm. A model of a set of spray-painting equipment and a canvas was added to an existing virtual robotic arm educational tool and used in an Introduction to Robotics class offered at Florida Gulf Coast University in Spring 2019 and Spring 2020. The class offered in Spring 2019 used the virtual arm but without the spray-painting feature while the class offered in Spring 2020 used the new spray-painting feature that was added to the virtual arm. Exam results show that 59.4% of the students that used the new feature scored at least an 85% on the corresponding differential movements exam question compared to only 5.6% of the class that did not use the added spray-painting feature. The differential movement exam question simply asked the student to produce a differential movements algorithm to move the arm with a specified velocity alone a straight line.

## Introduction

Introduction to Robotics is a common introductory robotics course generally covering the fundamental theory of robotics including robot kinematics, dynamics, differential movements, trajectory planning and basic computer vision algorithms commonly used in the field of robotics. This class mostly covers the study of controlling large heavy industrial articulated arms. Joint programming is the task of writing a program to directly control the robot’s joint motors as opposed to simply telling the robot’s controller where to move the arm. Ultimately, all robotic movements are performed by controlling the desired instantaneous acceleration, velocity or position of each joint motor. Joint programming involves all aspects of robotics covered in this class. Differential movements is a type of joint programming that moves the end-effector with precise velocity and has applications such as painting, welding, plasma cutting, and many others. Even though this class is very diverse and covers many different topics, differential movements, sometimes called velocity control, is covered in this class by over 50% of the instructors surveyed (Esposito, 2017). It involves an understanding of the relationship between the instantaneous velocity of each joint and that of the end-effector. The learning objective associated with differential movements, like joint programming, is to have the student learn how to create an algorithm that performs these movements. Before presenting the problem and solution statements, a more detailed explanation of differential movements and how the algorithm must compute them is presented.

### A brief introduction to differential movements

Differential movement is a type of motion control where the velocity of the end-effector is precisely controlled. It uses a closed-loop feedback system where feedback information from the joints sensors are used to close the control loop in order to achieve accurate tracking. It is used in applications such as painting, welding, plasma cutting, and other applications where the velocity must be controlled precisely. In contrast, a trajectory is a smooth movement from one point to the next. The controller uses an open-loop control system to produce a polynomial for each joint that tells the instantaneous acceleration, velocity, or position for each joint. However, the path polynomial is generated by providing a set of constraints such as initial and final position, velocity and acceleration, for example. Then, a polynomial with a sufficiently high order is derived that can satisfy all the constraints. The polynomial may not follow a constant speed since it is only designed to satisfy a small set of constraints. Differential movements on the other hand is like driving a car in that the controller observes the current velocity as feedback and applies new inputs as to maintain the correct desired velocity. Keep in mind that velocity consists of speed and direction.

The arm’s Jacobean matrix plays an important role in the derivation of the differential movements. The Jacobean matrix relates the rate of change of each joint to that of the end-effector. Since the joint velocities given the desired velocity of the end-effector are of interest, the inverse of the Jacobean needs to be computed. The Jacobian can be computed mathematically by determining the partial derivatives of the movement in every dimension of the end-effector relative to the movement of each joint. This Jacobian represents the movements of the end-effector relative to the world coordinate frame. Another form of the Jacobian can be computed using an algorithm with no need to compute any partial derivatives. It represents the movements of the end-effector relative to itself. Alternatively, the inverse kinematic equations can be differentiated and used in place of the Jacobian matrix. These equations will contain terms for the derivative of terms found in the rotational part of the forward kinematic equation matrix which will also need to be computed.

### Problem statement

Traditionally, universities tend to use real industrial robotic arms for this course. A survey of instructors for the Intro to Robotics course (Esposito, 2017) showed that 81.5% of the instructors indicated they use some type of hardware-based laboratory component. Today, commercially available industrial arms come with a controller that performs this joint programming, which, in fact, constitutes an obstacle in teaching. Students will need to bypass the arm’s included controller to gain direct access to the joint motors in order to perform any type of joint programming including differential movements. This is not desirable or possible as most modern robotic arm controllers include many safety features that, if overridden, void all warrantees and expose the educational institution to any liability resulting from accidents associated with the controller not being actively in control of the arm. It is unlikely any educational institution will allow such an activity. Furthermore, the use of large physical arms has many setbacks. They are expensive to purchase, require significant dedicated lab space, require technical expertise to maintain, and can be dangerous even with all its safety features. In fact, the survey of robotics course instructors (Esposito, 2017), shows that there are 34 different commercially available platforms that the participants indicated they use, none of which are the large articulated industrial arms that the class focuses on.

A second option is to use small light weight toy or educational arms; however, they generally do not offer variable speed motors or a way for the user to control the movement of each joint motor by specifying its instantaneous velocity. These smaller arms also do not have the dynamics of a large and heavy industrial arm making the implementation of differential movement algorithms trivial. A third option is to use a virtual arm simulator. This option works well for some aspects of the course but may not simulate the individual joint motors. That is, the robot model may not have the capability to move the arm by controlling the instantaneous velocities of the individual joint motors making it less effective in learning joint programming.

Regardless of the technology used, a challenge results when validating the correctness of a differential movement algorithm. The precision of the velocity control that is expected using differential movements cannot be observed with the naked eye. Therefore, to observe the correctness of the motion, one will need to perform some task that leaves a trace of the path such as painting, welding or performing some task that will leave evidence of correct or incorrect velocity control. There are no known virtual arms that contain these capabilities that a student can use to develop differential movements algorithms.

### Solution statement

One solution is to have a physical arm actually paint, weld or perform some real task. This solution has the drawback that it requires expensive materials to be consumed, equipment to be purchased, an environment to support the activity, and expertise in setting up the experiment and managing safety. A second solution is to use a virtual arm however there is no known educational virtual arm that exists that models the types of activities that require correct differential movements.

The solution presented in this paper consists of adding a model to an existing virtual robotic arm educational tool to simulate painting on a virtual canvas. The software tool presented here provides an opportunity for the student to perform joint programming in order to implement their differential movement algorithms using a virtual arm. That is, to properly control the arm, the student will need to provide the correct instantaneous joint velocity for each joint for every instance in time. The virtual arm will spray paint onto a virtual canvas leaving evidence of the correctness of the velocity control. The differential movement virtual arm software tool is an addition to an existing educational robotics virtual arm software tool that is described by Gonzalez & Zalewski (2017). Before presenting the solution in detail, some previous work is presented with comments to its effectiveness in teaching differential movements.

### Previous work

This tool is specifically designed to support teaching and learning essential concepts in an introductory robotics course. The Introduction to Robotics textbook, (Niku, 2011), was used to guild the development of this tool. The topics the tool supports are based on the topics covered in this textbook and include forward and inverse kinematics, the Denavit and Hartenberg (DH) parameter and frame placement convention, differential movements, trajectory planning including joint-level programming, and robotic vision. This material represents the fundamental theory behind controlling a robotic arm and it’s the theory that is used in creating the controller for a robotic system. Using a system’s controller to program its various robotic components is not part of this course and is actually a much simpler task to learn.

Our previous work on various parts of this tool (Gonzalez et al. 2015b; Gonzalez & Zalewski, 2016a, b), involve offering a way for the student to perform robotics activities without the need of an actual robotic arm. There exist a number of general-purpose robotic simulators both free and commercially available (GAZEBO Robot Simulation Software (n.d.); Robologix Logic Design (n.d.); Webots 7 (n.d.); Robotics Developer Studio (n.d.)). These tools are used for professional robotics research and related work as well as for educational purposes. The problem with using these general tools for teaching an introductory robotics course is that, first, there is a relatively steep learning curve needed to get sufficiently familiar with the tool before the student can use them for learning robotics. Our tool is specifically designed to allow a student who has never used the tool before to input the specifications of the robotic arm and get to the point where the student can move the links of the robot and adjust the viewing position within a few minutes. Second, actual robots are programmed using an included environment that uses a custom scripting language that performs all the inverse kinematics, trajectory planning, and joint programming required for the robot. While this is how real robots are programmed, it does not lend itself to learning introductory robotics since the logic in these preexisting software components is precisely what the student needs to learn how to create. This tool differs from these existing robotic simulation tools in that it is specifically designed to teach this specific course and therefore has a much smaller learning curve and does not do the work itself but rather supports the student while they do the work.

In the last decade, there have been in increase in robotic educational tools (Arocena et al., 2022; Santos, et al., 2019). Many are simulators design specifically for education however most are aimed at K-12 education and are inappropriate for university students. For example, Zhan et al. (2022) presents IRobotQ3D where student build the robot first then program the steps that will make it move. This tool combines a physical robot using the Lego Mindstorms kit and the IRobotQ3D simulator. Zhong et al. (2020) show that using the physical and virtual combination generally helps learning robot design but no significant difference was found in learning robot programming. The design of the robot is not with its D-H parameters but rather by adding wheels and other parts. The programming is not joint programming but rather a Scratch like language (Zhong et al., 2020; Maloney et al., 2010) that, while beneficial to learning standard procedural programming in a K-12 setting, it is not conductive for a university level course and not related to joint programming. Tijani et al. (2020), and Cheluszka (2019) present some possibilities of using inexpensive small educational robot kits in education however the programming component is aimed at the K-12 or technician and does not allow for joint programming. Nutakki et al. (2016) present a small robotic arm that can be controlled remotely via a web server. The web server has a camera that the student can see while they move the arm. While this allows for remote learning, the arm is a small toy arm with no dynamics and the web server does not allow for joint programming. Any remote lab setup where the arm is remotely controlled via the Internet will present a challenge for learning differential movements. The feedback loop needed to maintain precisely controlled velocity needs to update much faster than can be achieved through a web interface that is in the loop.

Peter Corke (2022) has developed a library of MATLAB functions (MATLAB Tool box for Robotics) and has made it freely available. This library is very popular but requires the student to write programs in MATLAB. There is no integrated development environment (IDE) associated with the library, so the level of programming is more extensive, and the investment of time needed to learn MATLAB, create the complete program and setup the virtual arm is much greater than using the tool presented here. This level of programming is not always feasible especially at institutions that do not have a software intensive program such as the Systems Engineering Program at Texas A&M International University, or in programs where this course is offered only as an elective. As an elective course that is not part of a robotics concentration, students are less likely to invest the time needed to appreciate the library from Peter Corke.

Another characteristic of Corke’s toolbox is that the philosophy of learning is different than the learning philosophy used to design the tool presented here. Tijani (2016), describes how.

Corke’s MATLAB toolbox can be used to allow the student to perform basic robotics activities. For example, they use the fkine() function to have the tool compute the forward kinematic equations of the arm provided. The jtraj() and ctraj() functions compute the joint and Cartesian space trajectory path. Our philosophy, however, is that a tool that performs these activities for the student is not as effective as a tool that supports the student in performing these activities. For example, our tool does not perform either of these functions but rather provides a programming platform where the student can program the arm, thus, requiring them to successfully perform the inverse kinematics and compute the trajectory plan. The end result is that the student must perform these activities, such as derive the inverse kinematic equations and compute a trajectory plan and use it in programming the arm. The tool does not perform these tasks for the student but rather only provides the programming platform that includes the virtual arm. The tool presented does provide some MATLAB functionality to support the student. For example, it can compute the inverse of a matrix needed in computing polynomial trajectories.

In fact, tools that preform the learning activities themselves rather than support the student in performing them are very common. For example, Sergeyev et al. (2017) describe a robotic simulation software called RobotRun that is free and open source that student can use however this tool simulates the arm’s controller as well. The intention is that students learn to program the arm using its controller. This is not the focus of this course or the tool presented here. In another example, Gonzalez et al. (2015a) describes a simulator for mobile robots, where the tool performs the motion planning. The user only enters some parameters that impact the way the tool performs these activities. The authors do claim the tool is to be used by the instructor in class and not to support the student directly. Manseur (2016) present a package of three tools, one of which, Inverse Kinematics Computations (IKC), computes numerical solutions to the inverse kinematic equations. The same tool allows the use of a virtual arm by providing it with a set of DH parameters much the same way it is done in the tool presented here. It also includes a symbolic processor for multiplying the individual transformation matrices to derive a set of forward kinematic equations. These are very helpful to the students however It does not appear that the tool allows the student to perform joint programming. In comparison the tool presented in this paper allows the student to program the robot’s joints individually to achieve the desired movements and provides an integrated development environment (IDE) supporting the programming environment.

All of these tools are useful for students that will focus their education towards robotics and will eventually need to work with very complex arms where these activities may be too difficult to do without such tools, however, in an introductory robotics course, the author believes it is better to provide a simple arm where the students can perform these activities themselves.

There is another group of software tools that are more aimed at supporting the student to learn. Robinette and Manseur (2001) present a tool that renders the arm given its D-H parameters. It is a web-based tool that one needs to interface via a TCP/IP socket connection. Once the arm is rendered the user can move the eye and see the 3D arm from different views. The presented tool also accepts its arm model by allowing the user to enter its D-H parameters and renders the kinematically correct arm using 3D graphics (Gonzalez & Zalewski, 2017). It also allows the user to move the eye and view the arm from different angles. Sanguino Mateo and Andujar Marquez (2010) present a tool that supports learning concepts related to forward and inverse kinematic equations and specifically deals with joint and Cartesian workspaces. The tool produces a reachability plot in a 3D Cartesian workspace by varying the theta angles. The presented tool supports learning forward and inverse kinematic equations by rendering the arm and allowing the user to move the virtual arm’s joints individually using sliders. Cakir and Butun (2007) present a tool that supports learning forward and inverse kinematic equations using quaternion algebra. Quaternion numbers are like complex numbers but work in four dimensions. The tool present in this paper does not support the use of quaternion algebra. Their tool does have some animated movement they refer to as trajectory planning but the user is not at all involved in the design of the trajectories only to specify the type of movement the tool is to use. The tool is not designed to support learning trajectory planning but rather is used as a way to support quaternion algebraic equations. In contrast to the presented tool, all of these tools support forward and inverse kinematic equations and the relationship between Cartesian and joint workspaces. They do not provide any support for learning joint-level programming or even trajectory planning. From the software point of view, all of the fundamental mathematical concepts including forward and inverse kinematic equations, transformation matrices, and the math involved in path planning are to support the ultimate goal of joint-level programming yet there is no known tool that directly supports this activity.

Differential movements used in moving the arm with a precise velocity is considered a type of joint level programming. Consider learning how to program using the Java programming language and not having access to a Java compiler. Many institutions use real robotic arms for this course however, using an actual industrial robotic arm will not allow student to execute joint-level programs either since, for safety reasons as explained earlier in this paper, their manufacturers do not allow direct access to its joint motors.

Many institutions also use small light weight arms to support this class. These arms can be printed on 3D printers or purchased for a small fee. Indri et al. (2013) use standard LEGO Mindstorm Kits while Schluse et al. (2020) use a virtual robotics lab that uses virtual reality to present a variety of robotics systems. This approach does not appear to be a robotic simulator that allows the student to program the movement of the arm.

Depending on the number of joints these arms may have, they may have sufficiently complex kinematics to make it appropriate for learning some areas of robotics however, because of their light weight, they do not have any significant dynamics. Without significant dynamics, differential movements become trivial and these arms not suited for learning this topic.

Another robotic platform that is commonly used for education are commercial robotic arms. These are real physical arms that may be larger and heavier than the small, lightweight arms presented above. These larger arms have a mass that allows a student to need to consider its dynamics. While these are good platforms for education they are large, expensive, require a dedicated lab space, expertise to set them up and maintain them, and may be dangerous. Additionally, they are not designed for education and generally do not allow the student to bypass the controller and gain direct access and control of its individual joint motors, a needed access to perform joint programming. One industrial arm, the Franka Emika robot (Haddadin et al., 2022) does allow for joint programming in an educational setting. It allows direct control over its joint motors while the controller still provides safety by observing the arm’s movements and overriding control when it gets into a dangerous situation. However, besides the relatively high cost, physical arms do not lend themselves to allow for remote or online education. In addition, to support differential movements, a physical activity will need to be added to this arm to record evidence of correct velocity control.

### Paper layout

The rest of the paper is organized as follows. Section 2 describes the solution in detail. It starts by presenting the design of the spray-paint model, followed by the student’s experience using this tool. Then the paper shows some potential educational activities that may be used with this tool followed by some sample canvas images produced with varying degree of differential movement correctness. In Section 3 the results of the assessments and their meaning are presented and in Section 4 concluding remarks are presented.

## Details of the solution

The feature that allows for the differential movement activity to be performed is the addition of a spray paint application to an existing virtual arm simulation software. Gonzalez and Zalewski (2017) describe the simulator they created that allows for joint programming activities. Joint level programming is required to implement the program that performs the differential movements. The spray-painting feature was selected over other applications such as welding or plasma cutting since it lends itself best for graphical output due to its painted canvas. The spray paint feature includes the painting canvas, the paint gun, and the addition of a set of user functions added to the joint programming language. The end-effector in this setup is the paint gun. To produce a painted canvas that shows evidence of correct velocity control, the student must move the arm with precise velocities and distance from the canvas. The differential movements must be correct. The painting model needs to be very accurate in order to capture even the smallest deviation in gun velocity or distance from the canvas. This model is described next.

### Design of the paint gun and canvas software

The simulation models the spray-painting activity by depositing virtual paint onto the canvas as the arm moves. Every time the clock ticks, the location of the end-effector over the canvas is used to determine which pixels on the canvas gets paint and how much paint.

The canvas is implemented using a two-dimensional array of pixels where each pixel is implemented as a structure with three bytes, one for each color, red, green and blue. White is the color when all three color components have their largest value of 255. Black is the color for all three values of 0. The canvas starts white so all three bytes in every pixel has the value of 255. As paint is deposited, the values of the green and blue are reduced leaving a light red. As the green and blue colors are reduced the resulting pixel color get darker red. Once the green and blue are both 0, the color is pure red. At this point depositing more paint is accomplished by reducing the red, the only color left. This makes the pixel color darker red towards black. As the red gets reduced the pixel gets darker and closer to black with the ultimate color of black when all three color components are zero. Black spots on the canvas generally indicate the arm stopped and while the gun was left painting.

How much paint each pixel gets is determined by using a convolution mask. When the clock ticks and the paint gun is on, the mask is placed on the canvas directly under the paint gun. Only the pixels under the mask gets paint. The paint flow is multiplied by the value in the mask to determine the amount of paint to add to the pixel below the corresponding component in the mask. Therefore, since the center of the mask has the highest values, the pixel under the center of the mask will get more paint. The pixels under the edges of the mask get less paint. The size of the mask is a user defined parameter. The mask is created by superimposing a polynomial centered over the mask.

**Fig. 1:**
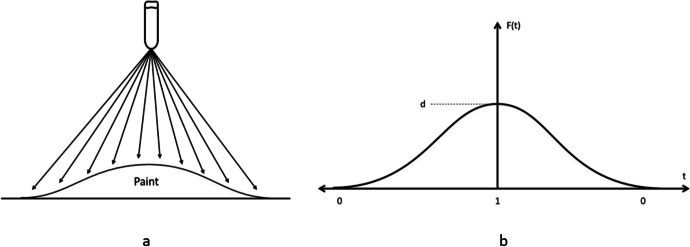
**a** The paint deposited on a perpendicular surface follows a curve with its highest point directly under the paint nozzle tip and decreasing outwards. **b** the curve used to model the paint deposited made by an equation and its mirror image

When the spray paint gun deposits paint on a perpendicular surface, paint distribution will follow a curve with the highest point being at the center and dropping towards the edges, see Fig. 1a. This curve was modeled with a 3^rd^ order polynomial going from the edge to the center. The complete curve is made by concatenating two such curves, one in reverse, see (b). The polynomial has to satisfy 4 constraints. The curve must be flat at both ends to have a continuous transition at the ends. Its second derivative must be zero at both ends. The polynomial must also go from zero at the edge to a height of d at the tall end. This gives two more constraints.

The following is the 3^rd^ order polynomial that was used to model the spray paint deposit along with its derivative.

$$f\left(t\right)={a}_{0}+{a}_{1}t+{a}_{2}{t}^{2}+{a}_{3}{t}^{3}$$

$${f}^{^{\prime}}\left(t\right)={a}_{1}+{2a}_{2}t+{3a}_{3}{t}^{2}$$

The constraints are:

1. The curve must be flat at the left edge, ${f}^{^{\prime}}\left(0\right)=0$.
2. The curve must be flat at the top or right edge, ${f}^{^{\prime}}\left({t}_{f}\right)=0$.
3. The curve must be zero at its left edge, $f\left(0\right)=0$.
4. The curve must be d at the top or right edge, $f({t}_{f})=b$.

The constraints give four equations that are used to solve for the four unknowns. Note, height *b* is an input parameter. The resulting polynomial is then simplified and parameterize to go from 0 at its lower end to 1 at its higher end. That is, instead of *t* going from 0 to ${t}_{f}$ it goes from 0 to 1. The resulting polynomial is:

$$f\left(t\right)=({3t}^{2}-{2t}^{3})b$$

where

$$t\in (\mathrm{0,1})$$

**Fig. 2:**
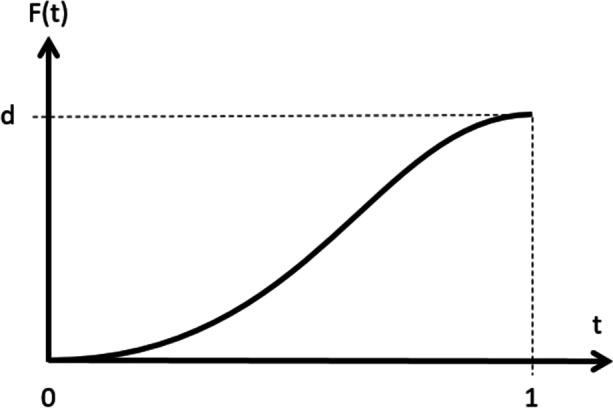
The curve $f\left(t\right)=({3t}^{2}-{2t}^{3})b$ with $t\in (\mathrm{0,1})$ used to form the mask

And *b* is an input parameter representing the height of the curve. See Fig. 2.

The mask was then created by shifting the curve in Fig. 2 above to the left by one unit then concatenating the mirror image of the curve to the right to form the curve in Fig. 1b also above.

Then, to compute the values of the pixels in the convolution mask, an algorithm was developed that visits each pixel in the mask. For each pixel, the distance from that pixel to the center of the mask in proportion to the total distance from the edge of the mask to the center is computed. This gives a number between 0 and 1. Then the paint polynomial gives the value to insert into that pixel using this distance. Finally, the mask is normalized by dividing every pixel in the mask by the sum of the values in all of the pixels such that the sum of all the pixels in the mask is one. Then to compute the amount of paint that gets deposited on to the canvas, each pixel in the mask is multiplied by the total amount of paint being deposited, then that value is added to the amount of paint already in the pixel on the canvas.

### Student experience

This section describes the experience the students gain from using this feature of the tool. Creating any type of joint programming program, including differential movements, involved writing a program in the Matrix/C programming language that was created for this educational robotics software. This new language consists of the basic “C” programming language with some MATLAB matrix instructions included. Esposito (2017) conducted a survey among instructors of the Introduction to Robotics course and found that the programming language used in the class is 62% MATLAB and 52% “C”. Gonzalez and Zalewski (2017) provide further details on this language.

The student starts by selecting an arm. The default arm works best for this activity however the student may create any arm they desire. The default arm is a simple two degree of freedom (DOF) with two revolute joint, (2R), planar arm that moves on the X–Y plane. Once the student selects or accepts the default arm, they then need to set up the canvas and the spray gun. From the main menu, they open the spray paint tab. There they select the canvas size, its position in the X–Y plane and its offset from the plane. The canvas is always perpendicular to the Z axis. Then the student must select the paint gun nozzle shape and size. The paint that is sprayed onto the canvas is confined to a rectangular region centered at the location of the gun. Within this region a second order polynomial determines the amount of paint to deposit on each pixel. This results in a very realistic looking paint application. The student selects the nozzle size which corresponds to the size of the region that gets paint. Then the student selects the paint flow. This is the amount of paint that flows out of the nozzle in a unit time period. The student must regulate the paint flow to correspond to the velocity that the arm moves the paint gun across the canvas.

Once the canvas and paint gun are setup, the student is ready to begin programming the arm to paint. The students will use the joint level programming feature, created by Gonzalez and Zalewski (2017) to write and execute their differential movement program. From the joint level program that they implement, they can turn on and off the paint spraying gun as the hand moves. The student must move the hand to an edge of the canvas. Then it must begin a straight constant velocity movement on top of the canvas towards the other edge. They turn on the sprayer as the movement starts and turns it off when the hand reaches the other side of the canvas. Then they must move the hand to the next row below and repeat the process adding paint below the previous row. They repeat until they complete painting the canvas. To achieve a uniform coat of paint, the hand must move straight across the canvas with a constant velocity. This is best achieved using differential movements. At the end of the program they can observe the painted canvas and see any imperfections such as paint streaks or gaps where too much or too little paint was applied. They can see alternating lines of dark and light paint where the paint flow and gap between the rows was not correct. They can look for uneven paint coverage where the student did not properly apply their differential movement theory which resulted in poor hand velocity control. They can see curved lines of darker or lighter paint where the hand did not move in a straight line. Like a real spray paint application, this feature produces realistic painting results that can be visually observed. Finally, the student can cut and paste their canvas into their project report to submit to the instructor. Sample canvas painted using this tool are presented later in this paper.

#### Setting up the Arm, Canvas, and Spray Gun

**Fig. 3:**
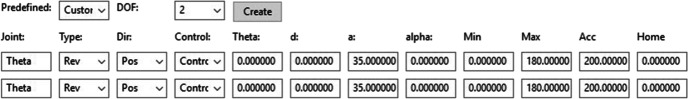
The arm creation tab. The user specifies the arm they wish to use in this window

Before the student can begin, the arm must first be created by entering information into the window shown in Fig. 3. For spray painting the default arm works well and the user may skip this step. The arm is entered by inputting its Denavit and Hartenberg (DH) parameters. These are in units that the student will decide such as millimeters or inches. The DH parameters is a set of 4 numbers per joint that specify the kinematic characteristics of an arm and are called theta, d, a, and alpha. With these numbers the motions of any realistic robotic arm can be represented. While the arm is rendered using stick figures, the arm is kinematically correct to a real physical arm with that set of DH parameters. Dynamic parameters can be entered as well. These include the range of motion and the maximum acceleration each joint can have. The students can also enter information such as if the joint is prismatic or revolute, a relation between an increasing angle or distance and the direction of movement on the arm, whether the joint is controllable or mechanically linked, and its home value.

**Fig. 4:**
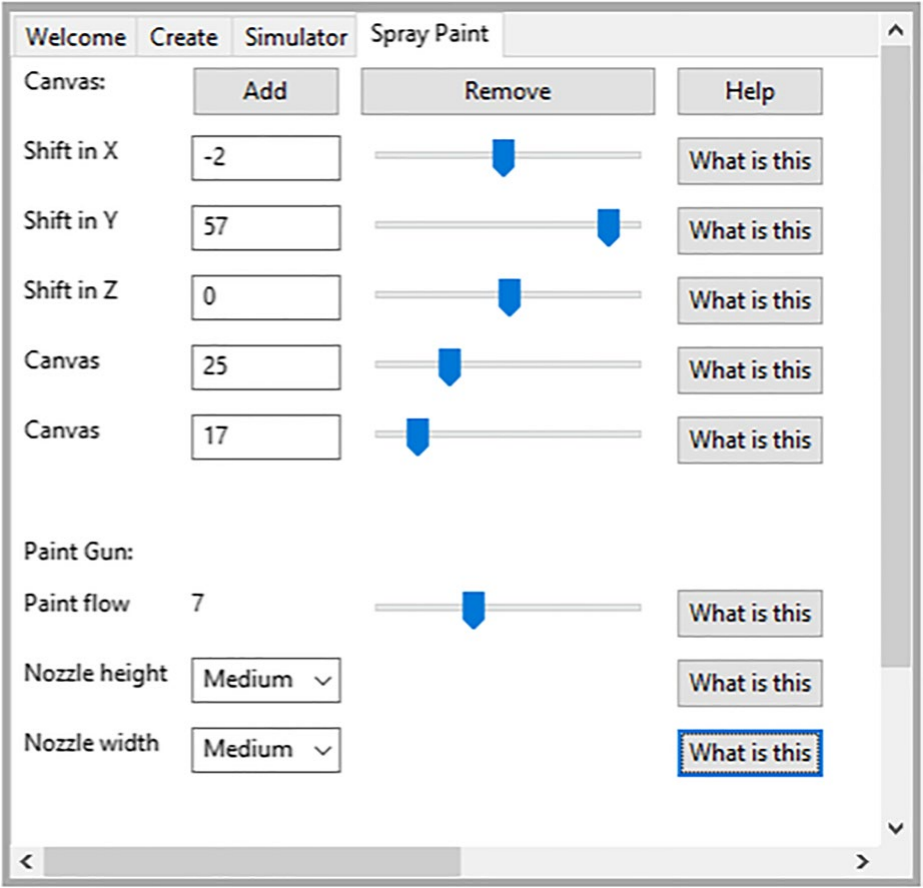
The spray paint setup window

Once the student creates the arm or decides to use the default arm, the paint equipment must be set up. The student can visit the Spray Paint window shown in Fig. 4. In this window the student needs to specify the dimensions of the canvas and its location as well as the nozzle specifications.

The student can enter the size of the canvas and press the “Add” button. At this time a box representing the canvas is added to the rendered arm shown on the left side of the screen in Fig. 4. The student can then move the canvas in any direction and change its size using the sliders while visually seeing the canvas size and location update.

Next the paint gun can also be set up if the default is not desired. The student can select the paint nozzle shape and size using the lower half of the spray paint window. Each dimension of the paint area can be selected to be small, medium or large. The nozzle can be tall and narrow, short and fat, or square with a large, medium, or small size. The paint flow is also set in this screen. The flow is the amount of paint that gets added to the canvas in each simulation clock tick.

#### Setting up the canvas rendering

**Fig. 5:**
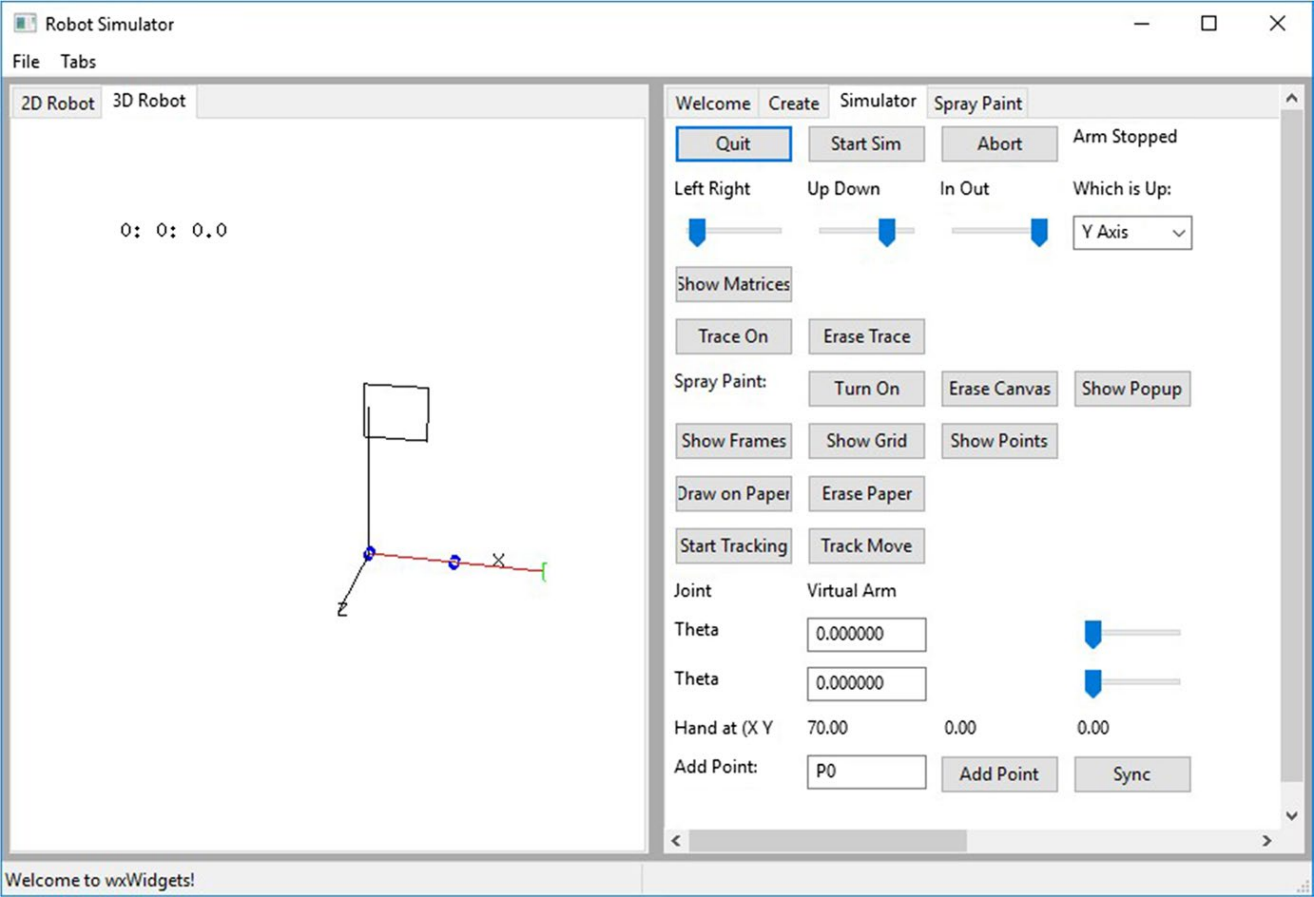
The pendent window. This window has the manual control of the arm and of the simulation

**Fig. 6:**
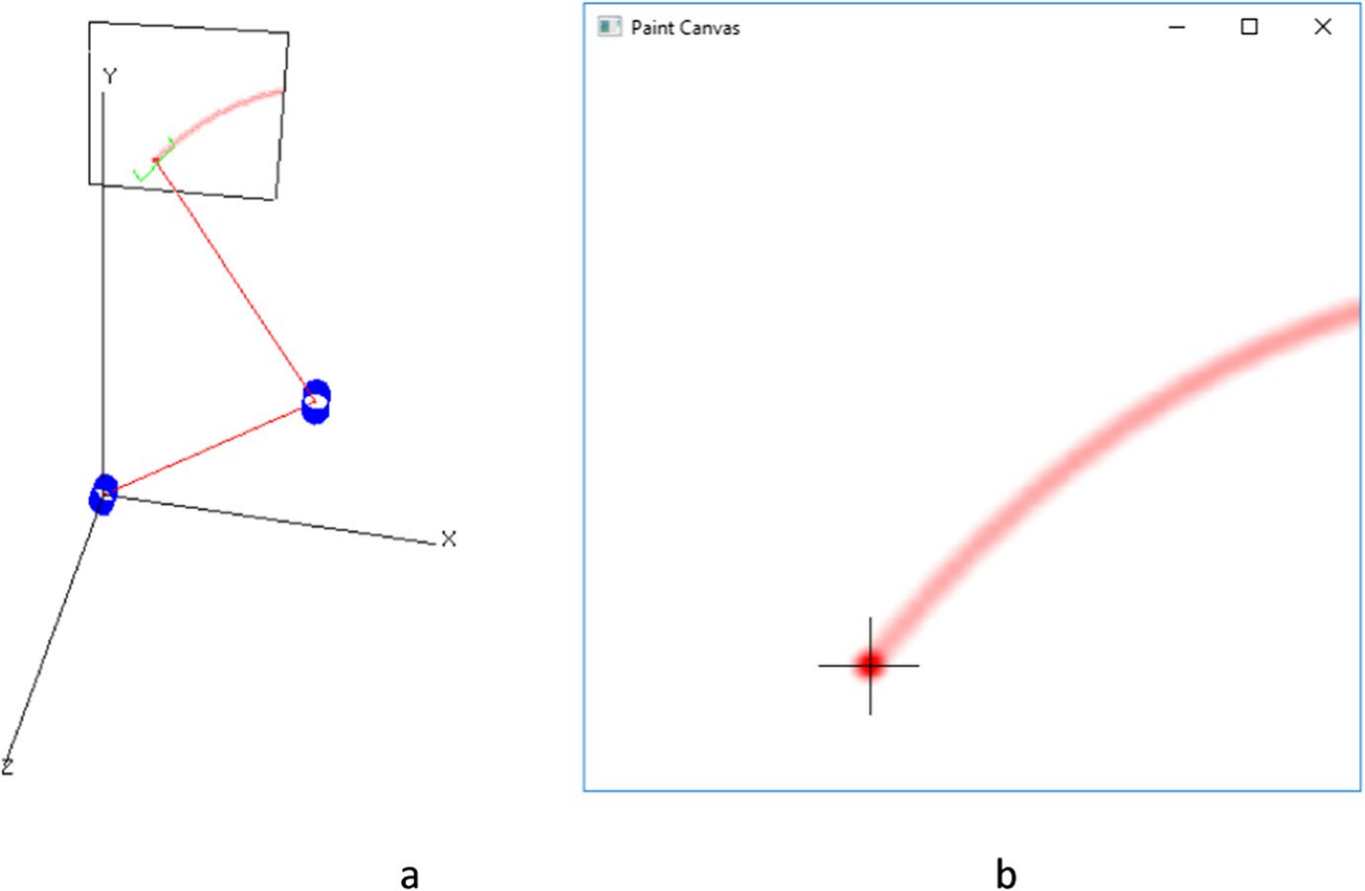
**a** The rendering of the canvas in the 3D image of the arm. **b** The rendering of the canvas in the 2D popup

The canvas is rendered in the rendering screen with the arm superimposed as in Fig. 5. Rendering the canvas in three dimensions (3D) requires that the location of each pixel in the canvas be transformed to the three dimensional coordinate system used to render the arm. This require multiplying the location of every pixel by a 4 X 4 transformation matrix to compute its location in the 3D image. Since this is a very slow process, only a low-resolution image can be rendered. To allow the student to see the canvas in its full resolution, a separate popup window is used to render the canvas in two dimensions (2D). See Fig. 6. The canvas is still rendered in the 3D image, Fig. 6a but at a much lower resolution and is used only to see the relative position of the arm over the canvas. The popup rendering, Fig. 6b does not show the arm superimposed but does show the canvas in its full resolution.

#### Programming the arm to paint

**Fig. 7:**
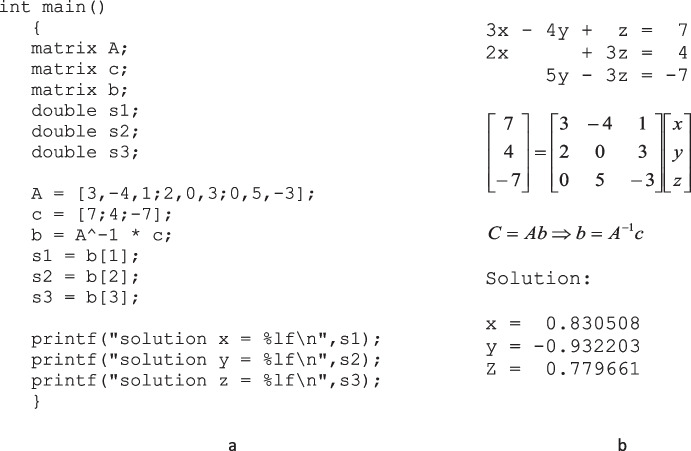
Example of a program written in the Matrix/C language designed for this tool. **a** The program computes the solutions to three simultaneous equations. **b** the simultaneous equations with the solution

Once the arm, canvas, and spray-painting gun are created and set up, the student can begin to create the joint control program. Joint programming is accomplished by writing programs in a language created for this tool. This language includes a subset of the “C” language commands with an added native type, matrix. It includes some MATLAB commands to facilitate matrix processing. The new native data type called “matrix” allows the programmer to perform direct mathematical operations on matrices much like what can be done with native types such as “int” and “double.” For example, using linear algebra, the program in Fig. 7a computes the solution of the simultaneous equations shown in Fig. 7b.

Because differential movement is a feedback based joint control method, the joint control program needs to be event driven. This includes the use of callback functions. The simulation engine of the tool uses a periodic interrupt to run the simulation of the arm. The simulation updates the arm’s dynamics once every clock cycle. The simulation engine has a list of callback functions that it must call at the end of every clock cycle. The student programmer must submit the name of their callback function to the timer interrupt service so it can get called at every clock cycle. They can do this by calling a system function and passing it the name of their callback function. This callback function is called every clock cycle after the simulation updates the arm’s dynamics.

In this function the programmer can call other system functions that provide it with the dynamic data of the arm such as the current position and velocity of the end-effector. Using this data, the programmer can implement their differential movement theory and compute a new velocity for each joint. The simulation engine will then use this new set of input velocities in its next clock cycle. In this method the student programmer can implement the feedback loop needed for the differential movement control.

In addition to programming the differential movement, the student must also program the arm to move from side to side painting row by row. The arm can be moved from side to side using the differential movement theory as it paints the canvas. Then, to move to the arm to the next row, the arm must move down a short distance with the paint gun off. Then it can proceed to move in the opposite direction to paint the next row. The complete program will include not only the differential movement theory but also the code to orchestrate the compete painting on the canvas.

Once the program is complete, the student can view and inspect the high-resolution two-dimensional canvas rendering for imperfections. Like a real painting robot, the canvas will show the result of the spray-painting program. For example, areas of darker and lighter paint may indicate the arm did not move with constant velocity, which is most likely due to the differential movement code not being correct.

### Educational activities

When programming a joint trajectory using joint programming, the student writes a program that computes the velocity of each joint for the duration of the trajectory. Following an open-loop model, when the program executes, the velocity polynomials computed are given to the arm’s controller all at once. At that time, the program ends and the virtual arm performs the movements. The arm actually moves after the program executes. The trajectory planning theory is about the computation of these velocity polynomial. However, the joint programming that is needed for implementing the differential movement theory, require that the program continuously execute in a feedback loop. Following a closed-loop, event driven model, the program must monitor the actual movement and correct for deviation in real time while the arm moves. This is needed for maintaining constant velocity while moving in an accurate trajectory. Table 1 summarizes the two types of educational activities.

**Table 1:** Two types of educational activities, trajectory planning and differential movements

Name	Trajectory planning	Differential movements
Movement goal	Move to a location smoothly to reduce energy and wear	Move with constant velocity
Application	Moving material, moving end effector to work location	Painting, welding, plasma cutting
Mathematical model	Velocity polynomials	Jacobean matrices
Programming model	Open-loop, sequential processing of instructions	Closed-loop, event driven, callback function called periodically
Feedback Loop	Not used	Control performed in feedback loop

The joint programming software was modified to allow for this type of feedback. A service was added to allow a student to submit a callback function to the simulation engine. This callback function is then called at every clock cycle. When the student’s joint program reaches the end, instead of terminating execution it goes into a sleep state. The callback function is executed every clock tick. In the callback function, the student must compute the Jacobian matrix, apply the proper differential movement theory and compute a new instantaneous velocity for each joint. This is a great exercise for, not only practicing their differential movements theory, but also for learning how to produce event-driven, real-time control programs using feedback loops with callback functions.

**Fig. 8:**
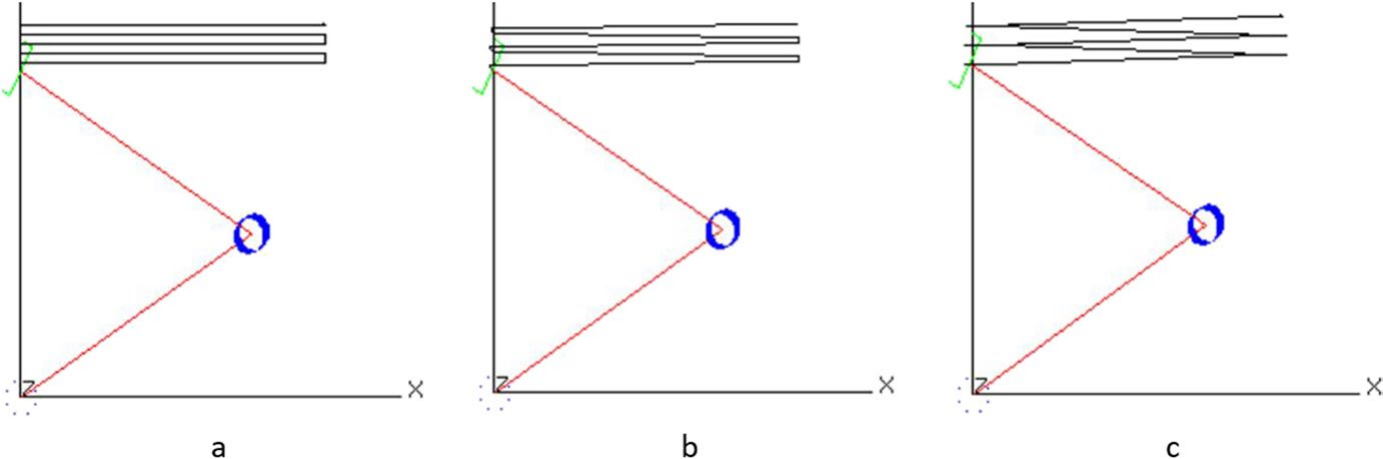
The path of the end-effector using three different speeds, **a** a speed of 1 unit per second. **b** a speed of 5 units per second and **c** a speed of 10 units per second

For example, one of the activities the student needs to perform is to determine the best speed for the arm. Since the program must be event driven, at each clock cycle, the program must compute the Jacobian matrix and use it to compute the next input velocity for each joint. This velocity will be used until the next clock cycle. The Jacobian matrix is a function of the arm’s current position. Therefore, as the arm moves, the Jacobian matrix become less accurate. The accuracy of the input velocity is dependent on the accuracy of the Jacobian matrix used. Moving too fast will cause the arm to move farther in a given clock cycle. This causes the velocity of the joint towards the end of the cycle to be based on a less accurate Jacobian matrix data. This may result in the arm deviating from its desired path and produce painted rows that are not straight. Figure 8 shows a trace of the end-effector as it moves with three different speeds. One can see the deviation between rows the faster the arm moves. On the other hand, moving too slow results in straighter lines but will take a long time to paint and may deposit too much paint on the canvas.

Being able to see that resulting painted canvas is the best way for the student to design the optimal speed as well as other parameters. The student has three other variables to control as well, the paint gun nozzle shape, the paint flow and the gap between the lines. The student is able to see the impact of their design parameters on the resulting paint that is applied to the canvas. The following section shows some sample canvas paintings produced by the software with varying parameters.

### Painted canvas samples

**Fig. 9:**
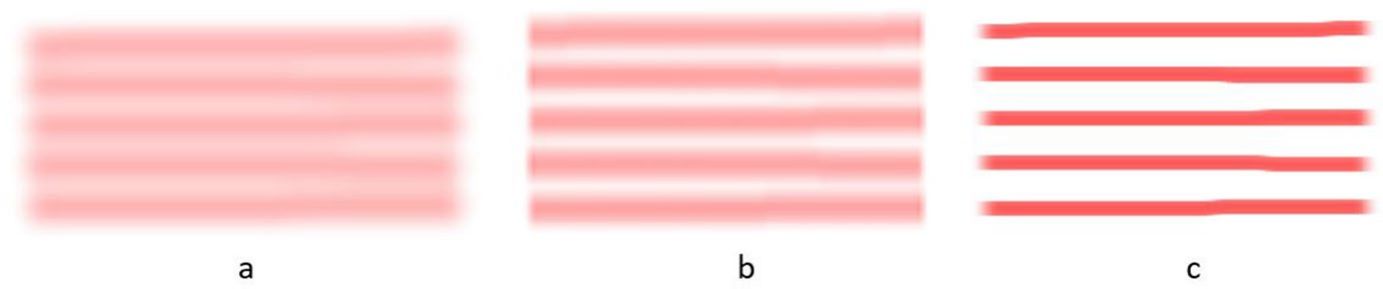
Three nozzle sizes using a flow of 7 units of paint and a gap of one unit between rows. **a** Using a large height and a large width. **b** Using a large height and a small width. **c** Using small height and a large width

The simulation models the spray pointing gun by depositing virtual paint onto the canvas as the arm moves. The canvas then shows the result of the movements of the arm. The following are sample canvas paintings for different program parameters such as the speed of the arm moving across the canvas and the size of the gap between the painted rows as well as the parameters of the painting gun such as the nozzle size and paint flow. All are in units of the arm. The arm used has a total length with the arm stretched out of 70 units. Figure 9 shows three canvas paintings using a flow of 7 units and a gap size of 1 unit and varying the nozzle size. The large gap size allows the rows to be clearly shown.

**Fig. 10:**
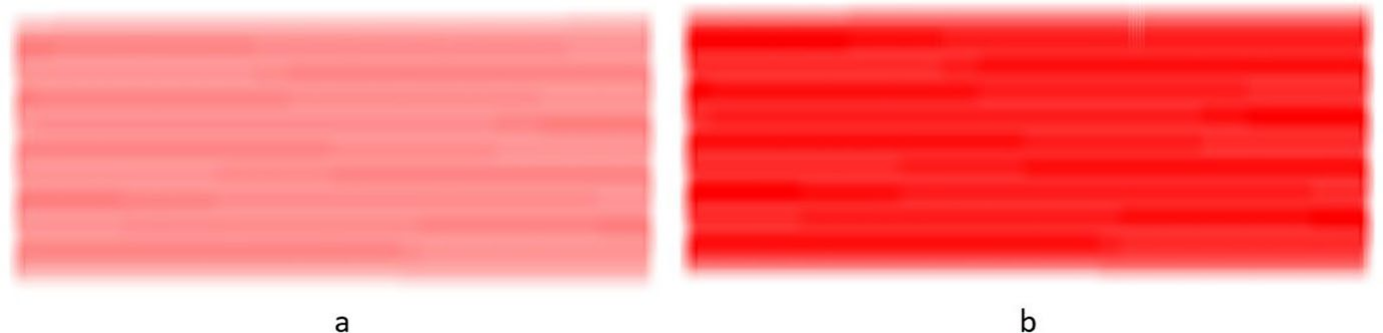
Two paint flow quantities using a gap size if 0.5 units, a nozzle with a large height and small width. **a** Uses a flow of 7 units of paint. **b** Used a flow of 14 units of paint

The following two painted canvases shown in Fig. 10 were produce using a gap size of 0.5 units, a large height and small width nozzle and varying the paint flow between 7 and 14 units of paint. Note the paint rows are still visible.

**Fig. 11:**
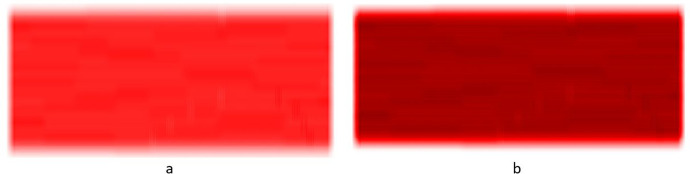
Two paint flow quantities using a gap size if 0.25 units, a nozzle with a large height and small width. **a** Uses a flow of 7 units of paint. **b** Used a flow of 14 units of paint

In Fig. 11 the nozzle gap size was reduced to 0.25 units. A large height and small width nozzle were used while varying the paint flow between 7 and 14 units of paint. Note in Fig. 11a the canvas is starting to look nice. In (b) too much paint is being deposited.

**Fig. 12:**
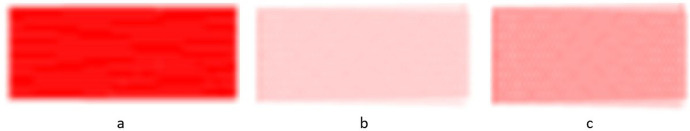
The flow and paint gun movement speed vary while the nozzle uses a medium height and width and the gap is 0.25 units. **a** Uses a flow of 7 units of paint and a speed of 1 unit/second. **b** Uses a flow of 7 units of paint and a speed of 5 units/second. **c** Uses a flow of 14 units of paint and a speed of 5 units/second

In Fig. 12 the nozzle size and gap size are fixed with a medium height and width nozzle and a gap size of 0.25 units. In Fig. 12a the arm moves slow with a speed of 1 unit/second while in Fig. 12b the arm moves 5 times faster with a speed of 5 units/second. Note the paint is lighter because moving five times faster results in one fifth of the paint being deposited. Then in Fig. 12c the paint flow was doubled to 14 units of paint. The canvas did not get sufficient paint however 14 units of paint is the maximum flow. The student will need to provide a second coat of paint or slow the speed of the gun. Additionally, in Fig. 12c the rows are not straight indicating the arm is moving too fast.

**Fig. 13:**
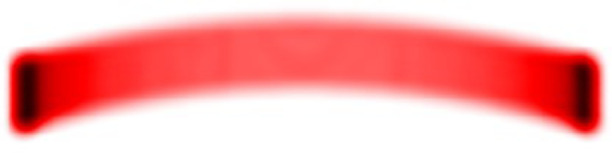
The canvas painted using trajectory planning theory as opposed to differential movement theory

Finally, a student may ask why trajectory planning theory cannot be used instead of differential movement theory which is more complex and requires the use of real-time control using a feedback loop. The most convincing answer is to try it and see. Figure 13 shows the canvas painted using trajectory planning. Note the arm does not maintain straight rows or constant speed.

These images show the feedback the students get from their algorithm and design. The software offers a realistic method of learning by allowing the student to design their parameters and algorithm following a model based on an actual application. Does this improve learning? The following section shows how well the software tool was at improving learning differential movements.

## Results

Two courses are used for comparison. One course was offered in the Spring 2019 where the feature was not used since it had yet been developed. The other course was offered in the Spring 2020 where the feature was used for the first time.

The assessment data presented in Tables 2 and 3 is based on question number three of exam two which asked the students to write a function that computes the instantaneous velocity for each joint given the desired speed and direction. They were asked to use the differential movement theory they learned in class. They were to assume they had access to a function that computes the Jacobian matrix. Both courses used the same question only with different numbers. Before the exam the students were also assigned a homework, which asked them to use the new feature of the software tool and write a program to move the arm with a paint gun and paint on a canvas as shown in Fig. 9 through Fig. 12 above. They were asked to adjust their algorithm and code until the arm was able to produce a nice-looking canvas. I 2020 the students were all given a copy of the tool to use on their own computer. In 2019 the students did not have access to the tool’s new feature and so they were only asked to compute the Jacobean matrix.

**Table 2:** Spring 2020 data where the new feature was used. The data is separated into two groups, those that mastered the differential movement concepts and those that did not. The threshold was a grade on the relevant exam question of at least 70%. This table shows the data for the students that used the new feature

Spring 2020 grade on exam question	Number of students in group	Average score on exam question (%)	Average score on whole exam (%)	Average score on the relevant homework (%)
$<70\%$	13	12.8	68.6	51.5
$\ge 70\%$	19	98.6	88.6	86.0
Total/Ave	32	63.8	80.5	72.0

**Table 3:** Spring 2019 data where the new feature was not available. The data is separated into two groups, those that mastered the differential movement concepts and those that did not. The threshold was a grade on the relevant exam question of at least 70%. This table shows the data for the students that did not use the new feature

Spring 2019 Performance on exam question	Number of students in group	Average score on exam question (%)	Average score on whole exam (%)	Average score on the relevant homework (%)
$<70\%$	14	17.1	58.0	78.6
$\ge 70\%$	4	78.0	83.8	100
Total/Ave	18	32.2	63.7	83.0

Before the painting homework assignment, the students were previously asked to use the tool’s joint programming feature to write code to move the arm using trajectory planning. Therefore, by the time they were asked to complete the differential movement homework, they were familiar with the Matrix/C unique language that is part of the tool.

The characteristics of the environment is important to understanding the assessment data. This course is offered once per year as a restrictive elective. The student must take 4 restrictive electives from a list of about 8 courses. They can also take some selected math or business courses in its place. Most students are seniors and taking their second course in a two-course capstone sequence at the time they take this Introduction to Robotics course. In the capstone course they are to work in teams and produce a software product for an external sponsor. This provides a challenge as the students tend to get very busy completing their large capstone project in the semester they typically take the Introduction to Robotics class. The students are also taking other courses that have a project component leaving them to become very busy.

In addition, during the Spring of 2020, Florida Gulf Coast University, had a sudden switch from offering classes face to face to full remote due to Covid-19. Unfortunately, the homework assignment involved in the assessment as well as the exam were issued and due during this course delivery change. It is believed that this had an impact on the student’s participation in the homework assignment and perhaps even in the overall exam performance. Each student received a copy of the software tool. They were able to develop their programs on their own personal computers.

The data in Tables 2 and 3 shows several conclusions.

1. Related to the exam, in the 2019 course, students did not use the presented feature and earned a weighted average of 32.2% on the differential movements question compared to a weighted average of 63.8% in the same question in the Spring of 2020 when they used the feature. This represents a 98% increase in the student grade. Since the differential movement exam question was 15% of the total grade in 2020 and only 10% in 2019, the students performed better and the question had more weight in 2020. This raised the average grade for the whole exam in the 2020 class although that class still performed slightly better overall than the 2019 class.
2. In the 2020 course, 59.4% of the students, 19 of 32 total, demonstrated that they can produce an algorithm to perform the differential movements of an arm compared to only 22.2%, 4 of 18 total, of the students who mastered the concepts in the 2019 class. The threshold to determine if a student mastered the concepts is 70%, where a student who performs at or higher than 70% should be able to implement a differential movement algorithm in practice albeit with some additional learning. If that threshold were to be increased to 85% instead, then 59.4%, 19 of 32, of the students would have mastered the concepts in 2020 compared to 5.6%, only 1 of 18, in 2019.
3. There is a correlation between the students who completed the homework using the software tool and their exam grade for the specific question compared to those who did not use the tool. Table 2 shows the average grade on the homework was 86.0% for the students that performed well on the exam question comparted to only 51.5% for those that did not in the Spring of 2020. This data shows that the students who completed the homework went on to do well on the exam question in comparison to those that did not complete the homework. A poor grade on the homework is generally due to the student not completing the work. The course’s grading policy is based on effort. That is, the students are given points based on the percent the project is complete even if it is not correct but shows good effort.

These conclusions are consistent with what is known about the impact software tools have on supporting students in learning how to create algorithms and develop programming skills. Computer programming homework almost exclusively always uses software that compiles and executes the student’s programs under development. It is well known that students benefit from the use of such software in developing their programming and algorithm development skills. Differential movements theory is used to develop velocity control algorithms. Therefore, learning this theory benefits from the use of a system that the students can use to develop, execute and verify their code in much the same way as learning how to develop algorithms and computer programs using a compiler.

## Conclusions

When learning how to control the movements of a robotic arm with precise velocities using differential movement theory, the student needs to implement test algorithms on an arm such that they can observe program correctness. The robotic arm needs to leave some type of trace that shows evidence of algorithm correctness since correctness cannot be verified by simply observing the movement of the arm with the naked eye. Using a physical arm with equipment to paint, weld or some other similar task is needed to record the precise movements of the arm but that has many drawbacks including the expense of the equipment, needing dedicated shop space and the risk involved with such activities. The use of a virtual arm with a model for an application that can be used to verity algorithm correctness solves these problems however there are no known virtual robotics arms that support this type of learning activity. The presented solution is to add a virtual spray-painting application to an existing virtual robotic arm simulator. The correctness of a differential movement algorithm can be observed by examining the resulting canvas after the arm applies virtual paint. This paper then presents some of the details in designing the spray-painting model along with some educational activities that can be used with the presented solution.

Data shows that students that used the virtual spray-painting arm to implement a program to perform a painting operation based on differential movements, performed better on the exam question pertaining to differential movements. The data shows that, 59.4% of the students that used the virtual spray-painting arm earned at least a grade of 85% on the relevant exam question which indicates they were able to produce software to move an arm using differential movements. In comparison only 22.2% of the student that did not use the tool earned a grade of at least 70% and only 5.6% earned a grade of at least 85%. The exam question simply asked the student to create a differential movement algorithm.

Future work includes giving the canvas the capability to have a curved shape to model painting a car or some curved surface. A more long-term goal is to add a feature to the virtual arm to support learning robot dynamics. In this scenario the joints of the arm will require different amounts of power depending on the position, velocity and acceleration of the arm as well as the load at the end-effector.

## Data Availability

Data sharing not applicable to this article as no datasets were generated or analyzed during the current study. However, the software created during and/or analyzed during the current study is available from the corresponding author on reasonable request.
